# Mapping mental health care services for children and youth population in Colombia’s Pacific: potential for boundary spanning between community and formal services

**DOI:** 10.1186/s13033-024-00626-w

**Published:** 2024-02-15

**Authors:** Sanne Weber, Francy Carranza, Juan Roberto Rengifo, Camilo Romero, Sergio Arrieta, Karina Martínez, Mónica Pinilla-Roncancio, Sarah-Jane Fenton, Germán Casas, Paul Jackson, Juan Pablo Aranguren

**Affiliations:** 1 Radboud University Nijmegen, Nijmegen, The Netherlands; 2 Universidad de los Andes, Bogota, Colombia; 3 University of Birmingham, Birmingham, UK; 4 University Hospital Santa Fe, Bogota, Colombia

**Keywords:** Colombia, Youth mental health, Interagency collaboration, Community health provision

## Abstract

**Background:**

Conflict and violence can impact on the mental health of children and young people, who are in a crucial stage of their personal growth. Not much is known about the provision of mental health care to young people in conflict-affected areas. Community-based care can be essential, as state-led services are often scarce in conflict contexts, like Colombia’s Pacific region where this research was conducted. According to the WHO, such care is ideally provided in the form of a network of interconnected services, offered by different actors beyond the formal health sector. This article describes the relationship between the formal and community mental health systems in Colombia’s Pacific region, and identifies ways of improving their interaction.

**Methods:**

Qualitative data were collected through 98 semi-structured interviews with community organisations, schools, international organisations and state institutions. These interviews aimed to identify the strategies used to promote young people’s mental health and the interactions between the different providers. Boundary spanning theory was used to analyse how different actors and forms of mental health care provision could coordinate better.

**Results:**

Community organisations and schools use a wide array of strategies to attend to the mental health of children and young people, often of a collective and psychosocial nature. State institutions offer more clinically focused strategies, which are however limited in terms of accessibility and continuity. International organisations aim to strengthen state capacity, but often struggle due to high staff turnover. Although mental health care pathways exist, their effectiveness is limited due to ineffective coordination between actors.

**Conclusions:**

To make sure that the variety of strategies to improve young people’s mental health effectively reach their beneficiaries, better coordination is needed between the different actors. Mental health care pathways should therefore integrate community organisations, while community connectors can help to manage the coordination between different actors and forms of clinical and psychosocial support.

## Background

Colombia has known a decades-long internal armed conflict between the state and different guerrilla and paramilitary groups, which caused over 450,000 deaths, 100,000 disappeared and 50,000 kidnapped persons, while over seven million people were forcibly displaced off their land [1]. Although peace was signed between the government and the principal guerrilla group, the Revolutionary Armed Forces of Colombia (FARC) in 2016, conflict is ongoing in many areas of the country. One such area is the Pacific region, which suffered severe violence during the conflict. High levels of violence and insecurity persist here, caused by conflicts between numerous armed groups because of trafficking in illicit goods and other illicit economies [2]. Several armed groups resort to the forced recruitment of people as young as 13, whereas forced displacement persists and social leaders are frequently persecuted and killed. The region faces high levels of poverty and lack of opportunities for employment and education in the Pacific region, with levels of poverty in Chocó and Nariño as high as 72% [1, 3, 4]. Violence and inequality also have a racial component, as the majority of the population is of Afro-Colombian descent with an Indigenous minority, groups which have faced historical racism and marginalisation.

What does it mean for young persons to grow up in a context that is characterised by both personal and structural violence? Experiencing or witnessing violence can impact on mental health and emotional wellbeing, which can eventually lead to long-term stress and physical problems [5]. Violence can have developmental repercussions for children and young people, as they are in a crucial phase of their personal growth [6]. Violence can produce changes to their perception of the world as a just place and to their quality of life. It also harms social networks and community trust. This in turn can affect their process of identity construction, whereas unaddressed traumatic experiences can increase behavioural problems, including aggression and suicidal ideations [7, 8]. Such experiences can also lead to substance abuse, which in turn can affect processes of learning, memory and decision-making [6, 9]. Conflict-affected children can have PTSD and depression, panic and anxiety disorders, sleep disorders, behavioural and conduct problems [10]. The degree of these problems varies considerably across conflict contexts, and depends among other things on the nature, degree and duration of the experienced violence [8].

Research in Colombia with children and youth victims has identified problems such as post-traumatic stress disorder (PTSD), substance abuse, suicidal thoughts, mood disorders, emotional problems such as withdrawal and isolation, and behavioural problems like aggression, violence, inadequate moral development, destructive behaviour, rule breaking and bullying, as well as lower educational attainment [6, 9–12]. The Pacific region, where our research took place, has the highest prevalence of psychosis among adolescents (12 to 17 years of age) in the country, while it also faces the highest prevalence of any psychological disorder (6.3% of all adolescents) and is among the three regions with highest exposure to traumatic events for adolescents [13].

Receiving adequate mental health support can help to prevent that experiences of violence have a lasting effect on young persons [14]. In Colombia, however, mental health care is unequally distributed [15]. Only one in ten people with mental health disorders receive adequate care and most psychiatric beds are offered in private hospitals which are financially inaccessible for a large part of the population [16]. Mental health services are over-centralised, with a gap between the central development of policies and their implementation at the local level [15]. This over-centralisation also diminishes the culturally sensitive perspective of mental health care, which is key to a majority Afro-Colombian population. Furthermore, 44 percent of institutions delivering psychiatric care are located in the four largest cities (Bogota, Barranquilla, Cali and Medellin) with only 2 percent in the poorest departments (La Guajira and Chocó). Similarly, 33 percent of psychological care is provided in the four largest cities, with only 3 percent in the poorest two departments [15]. Mental health problems tend to be higher among conflict-affected populations, among poor people and those living in rural areas. Socio-economic inequalities and political instability tend to be a risk factor for mental health difficulties, even when violent conflict diminishes [8, 17–19]. Adequate mental health care provision to these populations is therefore urgent.

Formal clinical services are sparse in the Pacific region [20]. Research in other conflict and disaster-affected regions has showed that access to community-based psychosocial services can help to rebuild social relationships and structures [14, 21]. Such rebuilt relationships and resources can help to prevent the potentially lasting effects of traumatic experiences and promote mental health and psychosocial wellbeing. The community is key, because of its social relations and geographical nearness and as it aims to facilitate recovery in and together with the community, to produce changes for that community [22]. According to the World Health Organisation (WHO), community-based mental health care is ideally provided as a network of interconnected services, consisting of mental health care integrated in general health, community mental health services, and services beyond the formal health sector, such as schools. Such community networks can improve the accessibility of care and decrease the stigma commonly attached to mental health care [23]. In this article, we describe to which extent such networks exist in Colombia’s Pacific region.

The WHO recognises that mental health is shaped by people’s social, economic, geopolitical and physical environment. Access to key social services can help to transform mental health, by enabling people to live a dignified life, find a sense of purpose and personal growth [23, 24]. Mental health thus goes beyond the mere clinical condition. The notion of psychosocial wellbeing is based on the interconnection between the individual and the society, including social relations and networks, the recovery of basic social needs, agency and citizenship. Psychosocial support thus combines emotional support with the recovery of life projects and social worlds, addressing both past trauma and current stressors, while considering the socio-political, cultural and historical context [25–27]. We take this holistic and community-based understanding of psychosocial support as the centre of our analysis.

Although mental health care support often focuses predominantly on adults, the WHO identifies the need for a life-stage approach [23]. Because of the difficult access to formalised mental health care provision in rural Colombia, it is important to understand which community-based services can compensate for this absence. Our research analyses how community and professional mental health services interact to support the mental health needs of children, adolescents and youth populations on Colombia’s Pacific Coast, analysing the main barriers and facilitators that affect the provision of services in an integrated mental health system. It innovatively applies boundary spanning theory to identify how different actors could be better connected to support the psychosocial wellbeing of children and young persons in Colombia’s Pacific region.

## Methods

This research forms part of a larger study, carried out by a multidisciplinary team composed of a social policy researcher, psychologists, development scholars, psychiatrists and a health economist. The study uses ecological systems theory [28] to understand the mental health resources for young people living in a conflict context in Colombia, by mapping the interactions of different actors and systems in the social environment of children and young people. Following on from a previous study of the macro system of public policy and legislation, the present research analyses the meso- and exo system, looking at the relationship between formal and community mental health systems at the regional level. Our research concentrated on the municipalities of Quibdó, Tumaco and Buenaventura, three major cities on the Pacific Coast, all with high levels of Afro-Colombian and Indigenous population. In these municipalities, we first used purposive combined with snowball sampling to identify community-based organisations which deliver different forms of mental health care support to young people. Based on the mapping exercise, organisations were given information about the project and invited for a semi-structured interview. We held 98 semi-structured interviews, all conducted in Spanish.

**Fig. 1:**
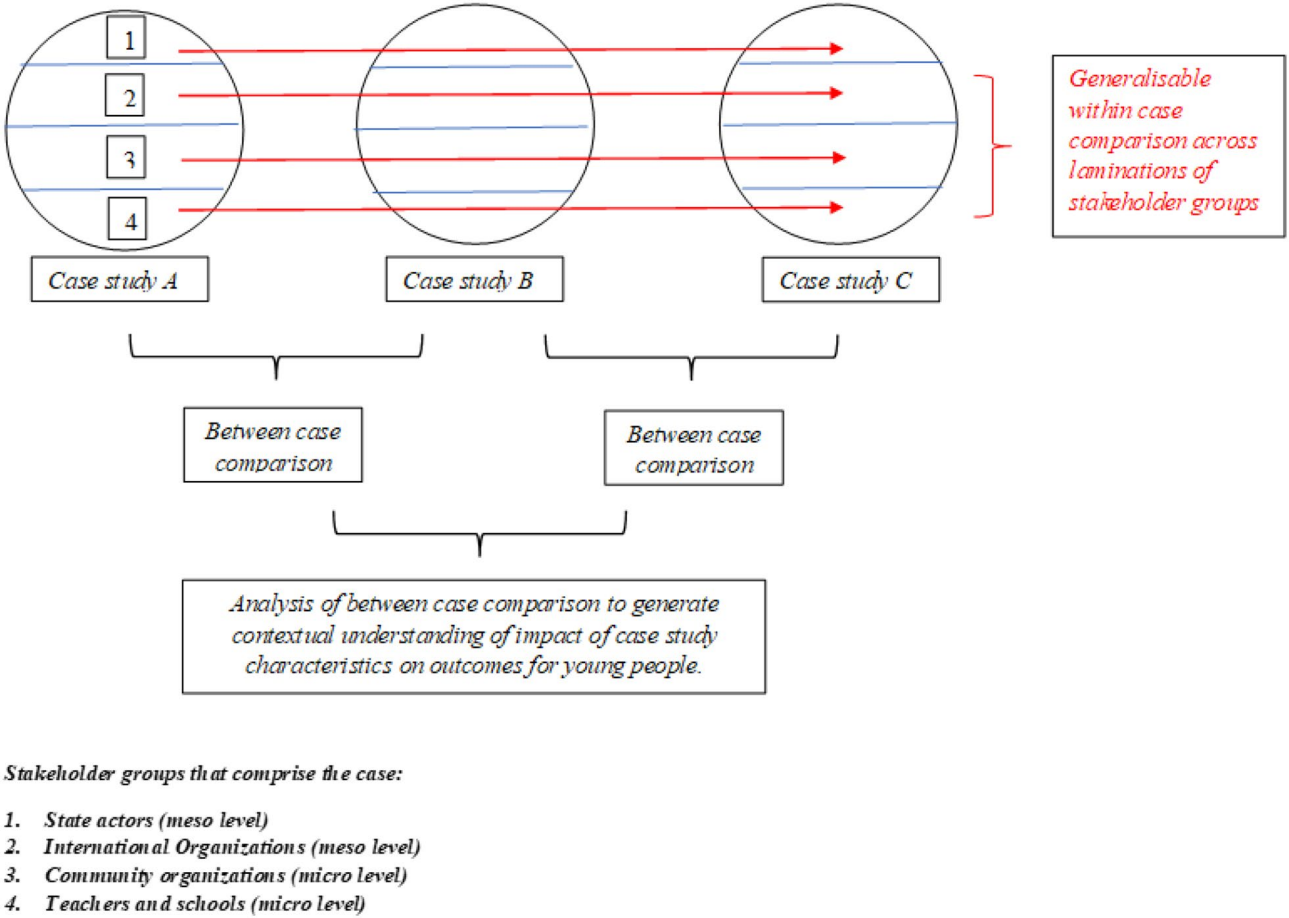
Research design for cross-comparative case analysis (adapted from [30, 31])

As illustrated in Table 1, we included four types of actors: state institutions, community organisations, international organisations and schools, although we focused particularly on community organisations and schools. Community organisations included organisations providing arts and sports activities for young people, as well as women’s organisations and organisations working with victims of the armed conflict. We complemented these interviews with interviews with state institutions, such as the regional and municipal Health Secretariat, Women’s Secretariat or Inclusion Secretariat, and international organisations. In this way, as Fig. 1 shows, we were able to undertake cross-case and between-case comparison, and achieve both coding and meaning saturation [29].

**Table 1:** Location and type of actors interviewed

	Buenaventura	Tumaco	Quibdó	Total
State institutions	1	1	4	6
Community organisations	17	10	14	41
International organisations	5	6	1	12
Teachers	22	7	10	39
Total	45	24	29	98

All participants gave their audio recorded verbal consent. The research received ethical approval by the Ethics Committee of the Universidad de los Andes through Acta No 1473 on 23 November 2021. Some interviews were conducted via Zoom and others in person. All interviews were held between January 2022 and October 2022, with three final interviews undertaken in May 2023. In addition, between March and October 2022 we held workshops in the three municipalities, where organisations and schools presented their youth mental health care strategies, followed by collective discussions guided by participatory methodologies on common experiences, challenges and benefits of their work. These workshops served to triangulate and complement the interview data.

The semi-structured interview guide aimed to understand the initiatives undertaken by the identified actors to promote young persons’ mental health. It was divided in four sections: the description of the actor’s initiatives and strategies; their understanding of psychosocial and emotional wellbeing; the limits and barriers they experience in their work; and the impacts they perceive. Interviews were transcribed verbatim and thematically analysed through inductive coding line by line, using NVivo (release 1.7.1). An initial codebook was developed by researcher SA based on the interview guide and an analysis of interviews in Buenaventura. This codebook was further developed by an interdisciplinary team of four researchers. Frequent discussions and joint coding of several interviews enabled the team to compare and reflect on disciplinary coding differences, and refine the codebook based on a shared understanding of the coding. The final codebook consists of eight themes, 43 codes and 46 subcodes. Coding consistency was checked and qualitative analysis undertaken by researcher SW. For this article, the analysis focused mainly on the codes related to psychosocial support strategies, limitations to the work of the interviewed actors, and the relationships between them.

To understand our coding framework and analyse the interactions between the different actors, in this article we use boundary spanning theory [30]. This theory originated based on lessons learned from the fragmented public service delivery in the United Kingdom’s decentralised health system. This forced institutions to find ways to ‘span the boundaries’ between them, by individuals who promote shared understandings and strategies through partnerships between different actors, institutions and sectors [31]. In Colombia too, the health system is designed to aim towards decentralisation. As a result, connecting different actors and levels proves challenging. Applying boundary spanning theory can therefore provide practical solutions. We use the concept of a care pathway, a boundary spanning object, to identify such solutions.

Our main focus in this research was on the support offered by schools and community organisations, since these are central in psychosocial support strategies and often act as first points of contact, while they are not included in formal health observatories [20]. In addition, we interviewed the most relevant state institutions. Although we managed to speak to key stakeholders responsible for mental health in the Pacific region, it proved difficult to obtain interviews. This might be related to the high rotation of staff in state institutions, as well as the lack of importance or even stigma attached to the issue of mental health among state health care providers. As a considerable part of our data about the state’s service provision is based on the views of community and international actors, further research on the perceptions and experiences of local-level state representatives is needed.

## Results

According to the WHO, community-based mental health care should function as a network of integrated services composed of formal health services, community mental health services, and services beyond the formal health sector, such as the school [22]. To identify the existence and functioning of such a network, we start with describing the strategies implemented by the different actors in the Pacific region, to then describe the connections between them.

### Psychosocial wellbeing strategies implemented by community organisations and schools

Community-based actors use a variety of strategies to promote psychosocial wellbeing among children and young people. Often these initiatives are not implemented by mental health professionals, but do address different elements of emotional and socio-economic wellbeing. The approach most used by both teachers (30.22% of data coded as psychosocial support strategies) and community organisations (16.44%) is the creation of safe spaces for listening, mutual support and the building of solidarity among persons with similar experiences. Such safe spaces, in which children and young people can talk freely about their concerns and take their minds off the violent context, also serve to identify risk factors, such as bullying, recruitment by armed groups, or (gender-based) violence, and orient and advise young people. They also serve, especially in schools, to improve harmony and mutual support among children and young people, and at the community level.

The often related and second most used approach among both community organisations (14.9%) and teachers (15.58%) is the use of artistic activities, including painting, music, theatre, dance, and storytelling. These activities help young people to describe and understand their experiences and express their emotions, in the absence of more formal therapy. These activities also distract young persons from their difficult situation for a while and prevent them from joining armed groups by finding other pastimes. Furthermore, art provides an indirect way of addressing emotions which is not seen as formal mental health support. It seems that arts are furthermore the method used most specifically for young people and children, whereas the other strategies, described below, are more general and also used for adults. An organisation from Quibdó explained the different benefits of arts-based activities:

We believe blindly that art is one of the best tools to transform societies. To make children conscious, to teach them their rights, and also to teach them that they have to comply with duties. We decided to create this festival to show that children, adolescents and young people from Quibdó are ambassadors of peace and reconciliation. Because through dance they break through invisible frontiers, they provide protective environments, they strengthen relationships and dignify their life projects (QUI_ORGCOM_13).

Arts-based methods are connected to another strategy, addressing and raising attention to human rights, which is common among community-based organisations (19.66%), but not schools (0%). The creation of murals or songs, for example, can send messages about human rights, thus combining emotional expression with activism. Given the situation of conflict and violence they operate in, it is perhaps unsurprising that community organisations are engaged in human rights-related activities, including the search for disappeared persons and the accompaniment of their family members, historical memory, broader processes of local peacebuilding, and sensitising children and young persons about their rights. These strategies are especially present among organisations in Tumaco, together representing almost 40% of their activities, whereas the use of more formal clinical strategies is nearly absent here, and artistic activities are also used less than in the other contexts. The organisations here seem to be more focused on direct responses to human rights violations and informal support strategies like mutual support groups, and less on broader artistic or cultural activities. In Quibdó, in contrast, we identified few organisations working directly on human rights-related issues.

Initiatives to promote livelihood strategies and skills training are also common for schools (11.21%) and community-based organisations (8.29%). In line with the WHO’s understanding of mental health as connected to social and economic conditions, participants frequently mentioned the lack of opportunities to study and work, as well as hunger and poverty, as reasons why young people experience mental health problems, and as risk factors for their joining armed groups. Livelihood and vocational strategies aim to reduce these risks, for instance through training on carpentry, agriculture, or handicrafts, as well as leadership, communication and other life skills. They help young people by giving them a sense of purpose. They are also frequently connected to artistic practices such as making and recording music or elaborating cultural projects. Although these initiatives are not necessarily directly connected to psychosocial support strategies, the idea is that helping young people to find their life projects prevents psychosocial problems by increasing their sense of control over their lives and future, occupying their minds and increasing their self-esteem.

There was also evidence of a considerable number of strategies among community organisations (8.65% of data coded as mental health strategies) directed towards recovering cultural traditions which were damaged because of conflict. Culture is considered a source of personal and collective strength and resistance against violence and discrimination. Community organisations—schools less so (only 2.8% of data)—undertake different activities to improve psychosocial wellbeing through the promotion of Afro-Colombian or Indigenous cultural traditions, especially in Buenaventura and Quibdó. Strategies include the use of medicinal and traditional herbs and plants, rituals and ceremonies, song and dance, as well as the performance of culturally specific mourning or burial rituals. These strategies combine the reconnection to culture and tradition with what organisations call ‘psychospiritual healing’ through recovery techniques from Indigenous and Afro-Colombian cosmovisions:

What we have done is connect the ancestral tradition of our authentic knowledge with that professional knowledge, because we consider that it is important to strengthen our roots, so therefore we make these connections. Right, a connection between Afro knowledge, Indigenous knowledge, that has really been very healing in those symbolic spaces that we have enabled (BUE_ORGCOM_13).

Some organisations also offer professional psychological support (13.51% among community organisations). Such support consists mostly of formal clinical therapy (10.29%)—which however seems to be inexistent among organisations in Tumaco—and to a lesser degree psychological first aid (1.38%) or the training of community agents, such as community leaders (1.84%). Many organisations however do not possess professional psychological or psychiatric expertise; their knowledge is mostly experiential: ‘My job is as a music producer, let’s say that one learns a bit of everything: one learns to be a psychologist, one learns to be everything, doctor, one needs to be everything here’ (QUI_ORGCOM_8). This is because in the Pacific region there are few opportunities to study these subjects. Most interviewees agree that there is a shortage of adequately trained professionals in this field. To some degree, international organisations fill this gap by providing training and mental health support methodologies to schools and organisations, as explained below.

Professional psychological accompaniment is also largely absent in schools (4.05% of coded data), where it is mostly limited to a first response to emergency situations. Schools instead often have a ‘psycho-orientator’ or social worker among their staff, who performs tasks like offering a listening ear to students; providing spaces and skills for conflict resolution, risk prevention and other social and communication skills; giving advice and sometimes workshops to parents; and accompanying the teachers with issues with students. Teachers however consider the lack of professional psychological expertise as an important barrier for addressing child and youth mental health. There is often only one ‘psycho-orientator’ for an entire student population of sometimes hundreds of students, whereas many teachers have not received training to detect or deal with mental health problems among their students.

Like I told you, there is only one psycho-orientator and sometimes it is a bit difficult for her to attend to that many students in one day. So, I don’t know, but I would like to have additional support from her, or that one as a teacher has the possibility to be trained to detect these cases (BUE_DOC_19).

For community organisations, this is a key barrier too. Some organizations indicate they do not possess skills for handling specific problems among young people, especially substance abuse. Others indicate that they lack capacities to deal with serious emotional problems:

We are not psychologists. We have something empirical and some group work. And if a person comes here who is perhaps very… very burdened, upset, super affected, who breaks in that moment there, then we will not have the capacity to help to balance or to manage the situation (BUE_ORGCOM_14).

In those organisations which do have social workers or psychologists, these individuals often indicate they are overburdened, as they are personally responsible for all the difficult cases:

This is all collapsing on me, because with the pandemic, the children, the family crises multiplied a lot. So, I was attending someone and another one called, and another one. Text messages, voices messages, videos… So, that was quite difficult, and on top of that the colleagues who called to request psychosocial assistance. No, at that time I experienced an impressive excess workload (BUE_ORGCOM_4).

Although from a psychosocial perspective, community-based approaches are essential for addressing emotional problems, including for their greater cultural sensitivity, there are limits when it comes to more severe cases of disorders or trauma, which require a clinical therapeutic approach. The examples given show that community organisations and schools tend to lack human and financial resources for these more severe cases. Here, a connection should be made with formal clinical mental health care provision through primary or hospital care. In fact, schools and organisations indicate they refer cases that require medication, clinical therapy or judicial accompaniment to state-led services. The next sections describe the difficulties in such referrals.

### Strategies by state institutions and international organisations

To understand how these community actors interact with formal mental health care providers, and form a community mental health network as envisioned by the WHO, we analysed service provision by the state and international organisations too. Compared to community actors’ broad and diverse approach, state-led strategies to support young people’s mental health are more limited. Their main approach is based on formal therapeutical strategies, including clinical therapy and psychoeducational activities (22.22%) and psychological first aid and primary response to emotional problems (14.81%), for instance in cases of gender-based or conflict-related violence. Several state institutions also explain that they have developed pathways for (mental) health support, which define the coordination between and actions by the different responsible institutions, including primary care centres, hospitals, the police, justice system and different state secretariats. A considerable amount of institutional activity (18.52%) is dedicated to disseminating these pathways—the next section describes this in more detail. In relation to less clinically oriented strategies, state institutions undertake sensitization activities on the need for mental health care and about risk factors (18.51%), such as talks in schools and communities about bullying and violence, or campaigns against substance abuse. In Quibdó, they also undertake livelihood and vocational skills training and peacebuilding activities (both 7.41%), although this might be explained by the fact that the institution interviewed here works with conflict victims, who often need support to rebuild their livelihoods.

When talking about their work with young people, community organisations often spoke about the difficulty they experienced in connecting to state mental health services. This was especially true in Tumaco, where comments about the limited work of state institutions composed 38.33% of the coded data about barriers to organisations’ work. Such complaints were less frequent in Buenaventura (15.62%), and Quibdó (20.55%). First of all, state institutions face a clear lack of resources and trained personnel. For instance, community organisations mentioned that in Buenaventura and Tumaco there is no permanent psychiatrist, but only periodic visits every two to four weeks. Therefore, the public hospitals are not always able to provide medication in serious and urgent cases. Sometimes they refer patients to mental health professionals in nearby cities, such as Pasto, Ipiales, Medellin or Cali, which makes services inaccessible to many people because of economic and logistical barriers. Several community and international organisations in all three locations mention the lack of understanding of the need for specialized mental health care among general hospital staff. Organisations also complain that the response of state services to mental health problems is slow and infrequent with therapy sessions only every two or three months, moreover not adapted to the specific situation of children and young people. In addition, there is a high level of turnover of staff working in public institutions, most likely related to the difficult context in the Pacific region with high levels of violence, poverty and inadequate infrastructure. This turnover increases work for organisations that engage with these institutions, thus complicating boundary spanning work:

There is a high rotation rate because people last only six months since they get tired quickly. So those we have sensitized in terms of gender, about Law 1257, about the care pathway, about intrafamily violence, after six months they are no longer there. And then other people come in who don’t know, who act as they like, who revictimize women, whose religious thoughts prevail over rights, and so this sets back many things (BUE_ORGCOM_16).

International organisations have similar complaints, and furthermore indicate their own problems engaging hospital staff: ‘really the interest, well, in mental health issues, keeps being something very stigmatised’ (BUE_ORGINT_1). Stigma and a reluctance to attend mental health problems or situations of violence is even more pronounced in the case of gender-based violence and other problems of women and the LGBTQI population, demonstrating the effect of a profoundly patriarchal system on health care provision.

It is important to mention that the interviewed state institutions did describe a variety of initiatives they undertake to promote young persons’ mental health, often focused on the community level, for example through collaborations with schools, neighbourhoods, families, and hospitals. Nevertheless, as the responsible persons in the health secretariats of the municipality of Quibdó and the Department of Chocó explained, there are obstacles towards implementing these processes on a continuous basis. Colombian regulations dictate that public officials are not allowed to have labour contracts of longer than a year. Designed to prevent corruption, the annual renovation of contracts in combination with lengthy bureaucratic procedures means that in practice many staff only have contracts for less than or sometimes only half of the year. This clearly affects the visibility and implementation of state initiatives, and as a result the state’s legitimacy. It also complicates more continuous coordination, which is key for boundary spanning.

International organisations play an important role in filling the vacuum left by the state. Like state institutions, they mainly focus on formal therapeutical strategies, including clinical therapy and psychoeducational activities (25.95%), training community actors on mental health (23.42%) and psychological first aid and primary response to emotional problems (9.49%). In relation to less clinically oriented strategies, they promote the creation of mutual support strategies and safe spaces for talking (17.09%) and undertake sensitization activities about the need for mental health care and risk factors (13.29%). Interestingly, in contrast to community-based organisations, the activities undertaken by international organisations rarely have an artistic or cultural element to them (3.8%).

A challenge with the work of international organisations, in contrast to state services, is that their coverage is not national. International organisations’ direct engagement with communities in the territories is also unevenly spread, which risks creating tensions, as a community organization from Tumaco explains: ‘Save the Children never came to my neighbourhood, Plan International never came to my neighbourhood […] There are over 63 organisations here in this territory and they focus on the same neighbourhoods, and that is unfair’ (TUM_ORGCOM_1). Although it is clear that international organisations simply cannot attend an entire population, better coordination between the different actors working in a territory could prevent some areas falling through the cracks.

### Fragile connections between youth mental health care providers

These four groups of actors each have specific approaches to mental health care services for young persons. This section describes how they complement or interact with each other—how they span their boundaries—towards a community-based mental health care network. Unfortunately, there are striking disconnections in the ideal model of integrated community-based mental health care. Most organisations and schools refer cases which require professional care to primary care centres or hospitals. In some cases—unfortunately the minority—such referrals are satisfactory, as for this community organization that has a very positive experience with a specific primary care centre (Entidad Promotora de Salud or EPS^1^)—but not with all:

For those (young people) who are in this EPS, the attention is magnificent. […] They are attended by a psychiatrist, an occupational (therapist), the psychologist. They are given medicine, are sent for laboratory tests […] But with the other EPS it has not been possible to obtain care (QUI_ORGCOM_11).

As described in the previous section, the support by most EPS and hospital services is less positively valued, and instead characterized by slowness or lack of response:

The difficulties with the institutions, sometimes in the referrals. There are children who definitely and absolutely, the assessment is that they do not need psychosocial treatment, but psychotherapy. So we have to refer them to the EPS where they indeed have a psychotherapy process. But this takes time. Here it takes over a month to obtain one of those appointments, and for someone who has a mild depression, they go there and they give him another appointment in another month. Well, that person needs what I call shock therapy, we need to be on top of them (BUE_ORGCOM_4).

Some organisations refer persons to private psychologists, but in many cases young people and organisations lack sufficient resources for this, while provision is limited, especially in more rural areas.

In the absence of sufficient public mental health care support, international organisations occasionally fill this gap. A well-known international organisation, for instance, used to deliver psychological support, including via telephone, in the territories. This support however terminated when this organisation ended its project in one of the research locations, as the Municipality’s Health Secretariat did not continue the service after it was transferred to them. A community organisation in Quibdó explains that communities seem to place more trust in international organisations:

Well, I believe there is more trust in international cooperation, or international institutions like UNICEF or the UN, and when something happens in the territory, without a doubt they have more influence at the national or international level… they are more listened to even than the local leaders, and they end up managing resources that the state institutions should manage. So trust is lost (QUI_ORGCOM_16).

In this way, even with the best intentions, the work of international organisations can have unintended negative consequences, by further damaging trust in state institutions or replacing them—a problem that boundary spanning work could help prevent. International organisations are increasingly aware of these risks:

Previously, international cooperation substituted the state activity, and that was a harmful action. […] So nowadays the focus of international cooperation is to complement the state action. […] Of course we are aware that often the municipal administrations do not have the resources to attend to this sort of situations, but at least they have to address them and start investing their resources, and this is where we come in. So the current model is to return the responsibility to the local administrations (TUM_ORGINT_6).

International organisations therefore frequently engage with state institutions to raise awareness on the need for and capacities to deliver specialized mental health care. Their appreciation of the receptiveness of state institutions differs, but they are unanimous about the need for such work: ‘What have we observed? That when there is accompaniment by international cooperation or by control organisms, such as the Ombudsman, the administrations move a bit more quickly’ (TUM_ORGINT_6). Like community-based organisations, international organisations signal that the high turnover of public officials—and in fact their own staff too—is a problem, especially given the short duration of international projects:

Well, the project is aimed at strengthening. We offer technical support in reality, for the state institutions. We always clarify that in all the spaces where we meet, we cannot replace, or take charge or responsibility of actions that the institutions need to comply with, because there are norms. The problem here is that the project ends and what we want is to leave capacity in place. But well, we see a constant rotation of officials in the territories. […] This doesn’t allow us to continue with what we have already advanced (TUM_ORGINT_5).

The focal point for mental health in Buenaventura’s health secretariat agreed that her institution collaborates with international organisations. This is not the case with community-based organisations, whose mental health strategies can in fact be essential for young people. This means that boundary spanning potential is lost.

### Potential for mental health care pathways?

An important tool to manage the connections and interactions between different actors in one or more related fields of practice, like the ideal model of community-based mental health networks, is a mental health care pathway. Care pathways are so-called ‘boundary objects’. They are multidisciplinary protocols or strategies to manage care by identifying chronologically the responsibilities of different actors. As such, pathways can enable shared understandings and working arrangements between actors from different sectors, including psychosocial work, clinical therapeutic services, child protection services, and justice or welfare systems. In our research locations, there are indeed care pathways. A community-based organisation in Buenaventura explains:

Yes of course, we have a pathway. In fact, it’s a pathway that we built together with the state institutions. Where the person would need to go first, who are those referring them, where are they, what is there role, right? To have clarity about the role of each institution and what they do in the care pathway. With the Prosecutor’s Office, with the justice sector […] with the health sector, the clinics, the hospital, the EPS. […] So there is work on a pathway and we have also lobbied, which is tiring, so that the institutions also have internal pathways. […] So this work that we have undertaken for 11 years with the Intersectoral Board has given some fruits, and we won’t say that the pathway is perfect or that it is always followed, but it has helped us so that the pathway more or less functions (BUE_ORGCOM_16).

Unfortunately, many other organisations emphasise that the pathway does not work. This might be because the entry points to the pathway are either the hospitals’ emergency units or the primary health system (EPS), which may not be the most effective starting points, as the previous sections have outlined problems in the health system to address mental health issues, due to lack of sensitisation and insufficient qualified staff. Some organisations are unaware of the existence of a pathway at all. This proves frustrating for involved state institutions, as an interviewee explained:

- Interviewee: I shared this pathway, I handed it over and showed everything via the beamer. And it seems illogical to me that they now say, in front of the students and everyone…
- Interviewer: That there is no pathway.
- Interviewee: That they don’t know it. It makes me sad… Wherever I go, I carry my leaflets, I carry the digital pathways. I express it and I show it and I explain it.
- Interviewer: Why do you think that the organisations do not use the pathway? What is the mismatch there?
- Interviewee: Perhaps it is not in the organisation or the delegated official. Sometimes what happens is that we go to meetings just to warm up the seats, but we don’t pay attention. And if we don’t take care, we later forget it. […] And we don’t have a replication of the information. Because usually we do this with teachers, or the coordinators of schools. They are invited so that they replicate the information…
- Interviewer: And they are not really replicating the information?
- Interviewee: Exactly (TUM_EST_1).

Other state institutions agree that disseminating information about the pathway and mental health care provision is a pending task: ‘We believe that it is a very powerful tool that we have, and if we disseminate it better, well logically more people can benefit from it.’ (QUI_EST_2). Coordination towards the pathway within and among state institutions must also be improved:

We have boards and boards and plans for everything. Meetings every day to think about all the problems. But one thing is when they are in the meetings, and another thing when they arrive here. People talk a lot about coordination. […] Here, everyone leaves the meetings with commitments, and they return after 8 or 10 days to the same meeting, and you will see that they return to the same commitments that were made (TUM_EST_3).

These anecdotes show that there is a willingness of public officials to take actions on youth mental health, but also problems with implementing the agreed actions and coordinating between—and within—different state institutions, civil society organisations and international organisations. Our data suggests, for example, that coordination between community organisations and schools is rare too, even though the psychosocial strategies offered by community organisations could help alleviate the work of overburdened teachers and ‘psycho-orientators’ in schools:

Here in Buenaventura there is a so-called mental health board. I was happy when they created this board, I said wow, they are finally considering the psychosocial from the public side. […] But now that there was this high suicide rate among young people, the only thing they did was a sort of radio spot saying, ‘talk to your family’. […] This board was so ineffective that various psychologists from Buenaventura started to connect among ourselves. We created a WhatsApp group to locate all of us and offer free psychological support to those cases and start processes in the schools. And what happened? There was no answer from the educational institutions. Because of the class times… Because of the times of whatever… I said, damn, the children are dying! (BUE_ORGCOM_4).

As a result, and confirming our previous research on systemic fragmentation of policy [15], the different actors involved in providing mental health support to children and young people in the Pacific region mostly work unconnected and in silos, each from their own disciplinary background and sector.

## Discussion

Our research has showed that different actors are actively providing essential and often culturally sensitive mental health support for children and young people in Colombia’s conflict-affected Pacific region. Community-based organisations and schools mostly provide psychosocial support activities, for instance promoting the creation of safe spaces and mutual support groups, artistic activities, actions to promote the defence of human rights and resist structural racism and marginalisation of Afro-Colombian populations, and vocational and livelihood skills training. Yet even though artistic strategies are widely used and can serve multiple purposes, they tend to fall short of providing clinical (or medical) support to work through the most severe cases of trauma among young people [32].

State institutions provide more clinical therapeutic approaches, but their response is often slow, partial and therefore inaccessible to most people, whereas state responses tend to lack the cultural sensitivity and more diverse forms of understanding psychosocial wellbeing that community-based organisations offer. International organisations have attempted to fill this gap by offering clinical psychological support, but because of the risk of replacing state services they increasingly focus on training and strengthening both state and community actors in relation to mental health for young people. These different actors thus work in distinct ways, which ideally would complement each other. There could be a role for national and regional policy to encourage greater boundary spanning and thereby influence practice in the territories. When community organisations and schools, who tend to be first points of contact for children and young people and are more closely connected to their cultural and socio-economic realities, identify mental health issues and provide psychological first aid, they should then be able to activate a mental health care pathway which initiates a smooth process of actions by different institutions providing a range of services.

It is increasingly recognised by authors critiquing the Western approach of understanding and addressing trauma, that in contexts characterised by long-term violence and inequality, psychological suffering is not necessarily caused by a one-off traumatic event. It can also be the result of long-term stressors such as poverty, lack of access to work, education or health care support, which cause individuals to feel little control over their lives [26, 33, 34]. This is why support for young people should not only address direct mental health needs, but also connect them to broader social services, such as housing and employment support [22]. In other contexts, such as Australia, work placements and vocational strategies have been effectively integrated into youth mental health programmes [35]. High-quality after-school programmes, providing tutoring, artistic or recreational activities are known to significantly improve young people’s psychosocial and academic development [36]. Furthermore, in our research there seem to be strikingly few initiatives explicitly targeting parents and families, while families are identified as a key protective factor for young people’s mental health [6, 8, 9]. Ideally, a mental health care pathway would therefore manage coordination between a wide range of actors, such as primary and potentially secondary health care services, child welfare institutions and other social services, community and international organisations, schools and even the police and justice sector. Other research [2] has however confirmed the difficulties we have identified in creating connections, mutual understandings and ways of working between formal and community actors.

Overcoming this challenge is what boundary spanning work aims to do. Boundary spanning theory describes the need for specialised individuals—boundary spanners—who enable collaboration and interaction between different organisational actors and services. This requires the creation of shared understandings of problems, needs and ways of working, the joint elaboration of plans of action and their smooth implementation through partnerships [30, 31]. Crucially boundary spanners can come from any sector, to facilitate effective working across two or more sectors. As our research shows, leaving such coordination to the general teams of each of these actors is unlikely to be effective, as each actor works from their own worldview and disciplinary strategy which is not always compatible across sectors. Moreover, most staff are too overburdened with their general workload for the intense process of negotiation and coordination which boundary spanning requires. This is especially the case with schools, which are accessible platforms for reaching young people, but where teachers are at risk of being overburdened with additional activities on top of their regular teaching load [8].

Enabling connections therefore requires a dedicated person (or team) with transdisciplinary knowledge to translate and connect ways of understanding and addressing mental health issues. A dedicated network manager or partnership coordinator can create and maintain relationships between actors with different interests and cultural practices [31, 37]. When working with communities, it can be beneficial to engage ‘community connectors’ who are familiar with the community and its dynamics, and can connect community members to relevant state services [38]. Coming from the community rather than state institutions, whose staff often come from outside the region and therefore quickly rotate, makes it more likely that boundary spanning work becomes more sustainable. Local leaders or community organisations can play an important role in this. They have legitimacy and trust among community and institutional actors, are aware of the financial, social, cultural and logistical obstacles that community members face when accessing mental health services, and are often first points of contact for young persons.

Care pathways, described in the previous section, can be a tool to facilitate boundary spanning work. Pathways are boundary objects, as they help to connect different social worlds and enable collaboration between services [39–41]. Nevertheless, our data showed that the existing pathways in the Pacific region are insufficiently known and used to actually serve this goal. Another obstacle is that their entry points are limited to the formal health system, which is often not accessed due to lack of accessibility or sensitivity, thus excluding a large part of the population from these pathways. Community organisations and schools, which tend to be closer—both geographically and socially—to the population should be included in the care pathways, especially as points of entry that can set the pathway in motion.

The creation of a pathway should not be seen as a goal in itself, but only as the start of a process of increased coordination. Other research has demonstrated that to actually make care pathways a useful tool takes long-term and intensive work, lobbying among the different institutions to create and use the pathway, and ongoing monitoring and follow-up work [40, 41]. The frequent comments about the lack of knowledge or stigma towards mental health among public health care professionals suggests that a continuous effort is indeed needed. To prevent local boundary spanners or community connectors from becoming overburdened, investment in terms of time, training and resources is needed [37, 42], also because our research showed that financial difficulties are among the most important barriers to the work of community actors. This is an important policy area for the Colombian state in its current efforts to reform the health system. Providing local municipalities with a dedicated budget, guidelines and training for inter-agency coordination through the creation of community bboundary spanning teams could help to take better advantage of the services already available in the territories, explicitly including the crucial work of community organisations. International organisations could support this process by training community connectors, preventing the current risk of duplicating or replacing state services, while building long-term local capacities. Both the state and international organisations could also train representatives of schools and community organisations on mental health first aid skills, which can increase awareness about mental health problems and thus improve access of (young) people to mental health support [43]. Together, these strategies can help ensure that children and young people receive adequate support to improve their psychosocial wellbeing, be it through clinical therapy, programmes for (artistic) expression and recreation, or vocational training and support.

To facilitate effective boundary spanning in and beyond Colombia’s Pacific region, future research should focus on the creation, dissemination and implementation of mental health care pathways, to understand better how the coordination between the different actors involved in implementing these pathways for children and young people at the local level could be improved. Furthermore, research should analyse specific elements of the pathway, for instance the role that vocational training can play in promoting mental health for young people in conflict-affected regions, how state-led provision can integrate cultural practices towards a more culturally sensitive approach, and how community and state-led mental health programmes can better engage families of young people.

## Conclusion

Our research has evidenced the diversity and richness of the work of community actors such as organisations and schools to promote the mental health of young people in Colombia’s conflict-affected Pacific region. Their work, mostly of a collective and psychosocial nature, can complement the clinical support provided by the state, which frequently falls short because of staff and resource shortages. Better integrating these different services could produce a more robust system of community-based mental health care. So far, connections between the different providers of mental health care to children and young people are fragile. The creation of boundary spanning teams, based on the training and funding of community connectors and the strengthening of mental health care pathways, could help create an integrated network of providers. Such a network could offer more holistic services to promote young person’s mental health.

## Data Availability

Available upon request from authors.

## Abbreviations

EPS: Entidad Promotora de Salud (Health Promoting Entity)
PTSD: Post-traumatic stress disorder

## Footnotes

1 The EPS are private intermediaries of public resources for health provision, mostly used by middle- and upper-class citizens. The different EPS have very different functioning and performance standards. Sometimes, EPS subcontract with private providers, although in most cases provision goes through public hospitals, especially in serious cases and emergencies. Poorer classes receive health care through the public provider SISBEN.
